# Mandibular first premolar with five root canals: a case report

**DOI:** 10.1186/s12903-020-01241-0

**Published:** 2020-09-10

**Authors:** Ming Zhang, Jian Xie, Yan-huang Wang, Yan Feng

**Affiliations:** 1 Fujian Key Laboratory of Oral Diseases & Department of Endodontics, School and Hospital of Stomatology, Fujian Medical University, 246 Yang Qiao Middle Road, Fuzhou, 350002 China; 2 Stomatological Key Laboratory of Fujian College and University & Department of Preventive Dentistry, School and Hospital of Stomatology, Fujian Medical University, 246 Yang Qiao Middle Road, Fuzhou, 350002 China; 3 Fujian Provincial Engineering Research Center of Oral Biomaterial & Institute of Stomatology & Department of Preventive Dentistry, School and Hospital of Stomatology, Fujian Medical University, 246 Yang Qiao Middle Road, Fuzhou, 350002 China

**Keywords:** Anatomic variation, Dental operating microscope, Root canal system, Endodontic treatment, Case report

## Abstract

**Background:**

Understanding the anatomical morphology of the root canal is key for successful root canal treatment. The aims of this case presentation are to report a unique case of root canal treatment involving five root canals in the mandibular first premolar and to highlight the importance of variation in root canals of mandibular first premolars in clinical practice.

**Case presentation:**

A 25-year-old male with intermittent pain in relation to the lower right posterior teeth over 3 weeks was diagnosed with symptomatic pulpitis in tooth #44. Four root canals were found, including mesiobuccal, distobuccal-1, distobuccal-2, and distolingual roots, and the Mtwo rotary system was used for root canal preparation. The four root canals were filled after 2 weeks, when a fifth canal was found, located in the buccal cavity. The fifth canal was confirmed to be the mesiolingual root canal by cone beam computed tomography (CBCT) and was found to be curved. After completion of the root canal filling, CBCT was performed, and a three-dimensional root canal image was reconstructed. After 1 week of observation, the tooth was repaired using composite resin filling.

**Conclusions:**

This is the first case presentation of a fifth canal of the mandibular first premolar and advances our understanding of variations in the anatomy of the mandibular first premolar. This case report provides a reference for the treatment of mandibular first premolars.

## Background

The purpose of root canal treatment is to clear pathogenic microbes and infected pulp in the root canal, prevent it from producing toxic products, and protect the periapical tissue [1, 2]. The presence of root canal variation increases the difficulty of this treatment [3–5]. It has been reported that 42% of retreatment cases are due to missing canals [6]. Therefore, there is great clinical significance for dentists to master the morphological characteristics of root canals. Branch root canals are common morphological variations in root canal systems, whose morphology can present either as one or more small lateral branches from the main root canal or as two equally large bifurcated root tips on the apical segment [7, 8].

The anatomical variations in the root canal system of the mandibular first premolar are known [9–15]. For example, in an Egyptian population, it was shown that 96.8% of root canals of mandibular first premolars present as a single canal, and 3.2% present as two canals [16]. A review reported that approximately 97.90% of mandibular first premolars had a single canal, 1.8% had two, and only 0.2% showed three canals, while very few showed more than three canals (< 0.1%) [17]. The purpose of this case presentation is to present a rare case of a first mandibular premolar with 5 root canals and their root canal treatment.

## Case presentation

The patient, male, 25 years old, conscious, with no underlying disease, was admitted to our hospital in April 2016 due to intermittent pain in relation to the lower right posterior teeth over 3 weeks. Clinical examination found acceptable oral hygiene and deep distant proximal and occlusal caries penetrating the pulp chamber on tooth #44. Compared to the same tooth on the opposite side, tooth #44 showed slight knocking pain, sensitivity on the electric pulp test (Digitest, Parkell, New York, USA), and a positive response to a heat test with a tooth glue stick. There was no evidence of swelling or sinus tracts on the alveolar mucosa. Diagnostic X-ray showed that tooth #44 had distant proximal and occlusal caries penetrating the pulp chamber. The periodontal ligament was found to be slightly widened. Preoperative periapical radiographic examination revealed no apical radiolucency in association with tooth #44. According to the above results, we diagnosed the patient with symptomatic pulpitis in tooth #44. Root canal therapy (RCT) was proposed.

**Fig. 1:**
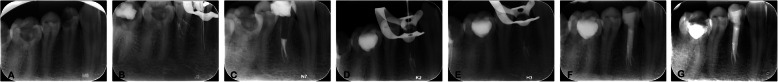
Preapical radiographs of tooth #44. **a** The lower third of the root canal of the first right premolar had branches; **b** Working length radiographs of four root canals of the tooth were measured; **c** Four root canals were filled; **d** The missing mesiobuccal root canal was found and measured; **e** Radiograph showing obturation of all five canals; **f** Recall observation X-ray 6 months later; **g** Recall observation X-ray 48 months later

Because of the complex shape of the patient’s root canal, the treatment process also included repeated exploration of the root canal anatomy. In the early stage of root canal treatment, X-ray showed that the branch canals were in the lower third of the root canal (Fig. 1a). Four canals were found under a dental operating microscope (OMS 2350, ZUMAX, Suzhou, China). During root cleaning and shaping, a fifth canal was also found later (Fig. 1).

Under local anesthesia, the caries were removed, and the top of the pulp chamber was completely exposed. The tooth was built by composite resin restoration. The treatment was performed under rubber dam isolation using a dental operating microscope. Four root canals were found at the lower third of the root canal, which were mesiobuccal, distobuccal-1, distobuccal-2, and distolingual roots. The orifices of the mesiobuccal, distobuccal-1, and distobuccal-2 root canals were located at the same level. The distolingual root canal was located on the distal wall of the tooth, 2 mm above the level of the other canals, approximately 1 mm away from the distobuccal-1 root in the buccal direction.

The initial working length of the root canal was determined by diagnostic X-ray and an apex locator (Propex pixi, Dentsply, Ballaigues, Switzerland) [Fig. 1b]. The #8 and #10 K-files (K-FILES, MANI, Tochigi, Japan) were used to dredge the position that was 3 mm less than the initial working length. The upper sections were opened with IntroFile (FlexMaster, VDW GmbH, Munchen, Germany). The orifices of the root canals were trimmed using ET18D (ET18D, SATELEC, Merignac, France) under a dental operating microscope. Straight paths were established. An Mtwo rotary system (Mtwo, VDW GmbH, Munich, Germany) was used for root canal preparation. The lower section was prepared to a #25/0.06 taper. A total of 15 ml of 2% sodium hypochlorite was used to irrigate every root canal during preparation. After preparation, an ultrasound system (P5 Newtron XS, SATELEC, Merignac, France) was used to activate sodium hypochlorite, and 15% ethylenediaminetetraacetic acid (EDTA) solution was used to irrigate the root canal for 1 min. After root canal desiccation, calcium hydroxide paste (TYPE I, GUANYA, Wuhan, China) was filled in and temporarily sealed by zinc oxide (Ceivitron, Dongquan, Taibei).

**Fig. 2:**
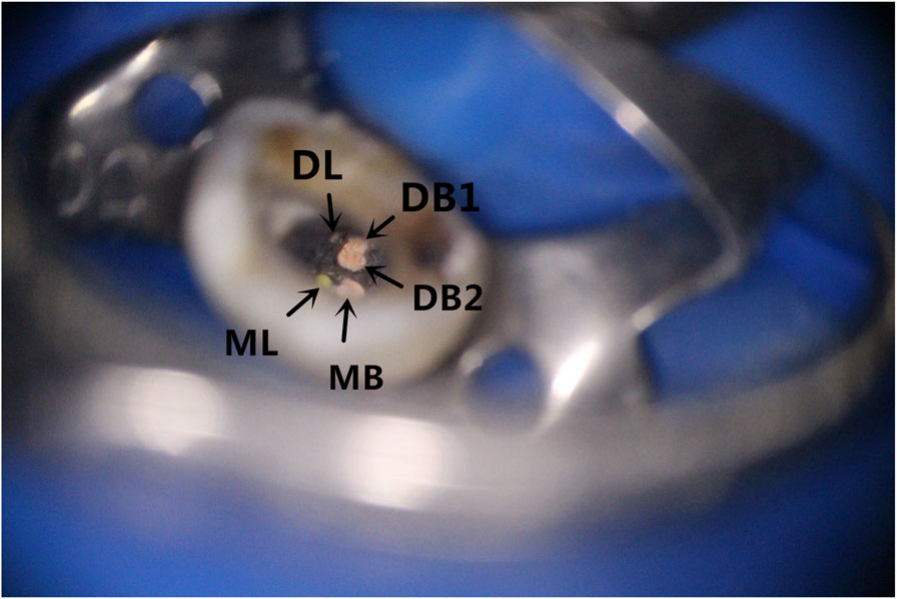
Orifices of the five root canals of tooth #44 with obturation under the microscope. Mesiobuccal canal (MB), distobuccal 1 canal (DB), distobuccal 2 canal (DB2), mesiodistal (ML), and distolingual (DL)

The tooth was re-examined 2 weeks later. Four root canals were filled with large taper gutta-perchas (RECIPROC, VDW GmbH, Munich, Germany) and root canal sealer (AH plus, Dentsply, Konstanz, Germany) using a vertical condensation filling system (Elements, SybronEndo, Glendora, CA, USA) (Fig. 1c). After filling, it was found that there was a fifth canal on the buccal side. The fifth canal was confirmed to be the mesiolingual root canal by CBCT (GIANO, NewTom, Verona, Italy), and it was curved. This root canal was located at the #8 K-file, and the length was determined by the apex locator (Fig. 1d). The root canal was prepared with the Mtwo system and temporarily sealed with zinc oxide. The fifth canal was filled with large taper gutta-perchas (#20/0.06) and AH Plus sealer using a vertical condensation filling system. After filling, the microscope (Fig. 2) and the X-ray image clearly showed that the five root canals were fully filled, and no obvious abnormalities were found on the tips of the roots (Fig. 1e).

**Fig. 3:**
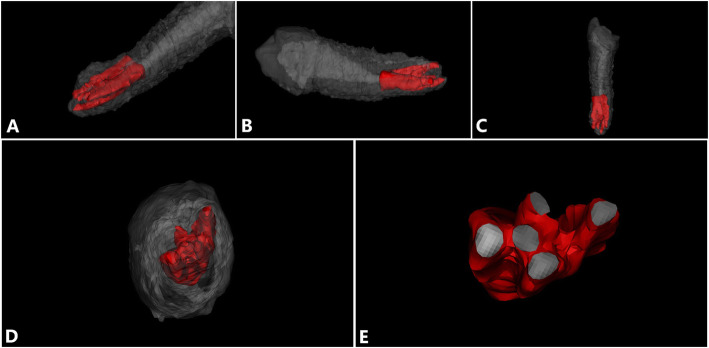
3D reconstruction of the root canal of tooth #44 after filling. **a-d** 3D reconstruction of teeth from different angles; gray is the tooth tissue, and red is the root canal tissue. **e** A cross section of the root canal at the apical area

After completion of the root canal filling, the CBCT examination was performed, and the three-dimensional root canal image was reconstructed (Fig. 3). After 1 week of observation, the patient showed no spontaneous pain, no pain upon hot or cold stimulation, and no obvious abnormalities in the buccal or lingual mucosa. The patient refused crown repair. The tooth was repaired using composite resin (Filtek Z350 XT, 3 M ESPE) filling. Six months later, X-ray showed no apical radiolucency in tooth #44 (Fig. 1f). After 48 months, the treated tooth #44 showed no secondary caries, no fracture, and no apical radiolucency (Fig. 1g).

## Discussion and conclusions

The root canal of the mandibular first premolar has great anatomical variations, far more than the second premolar of the mandible [18], as documented in the literature [9–15]. However, to date, most studies report cases with two to three root canals. Cases of four root canals are rare [19], and cases of five root canals on the mandibular first premolar have not been reported. Studies have shown that due to root canal variation, the failure of root canal treatment for mandibular first premolars is as high as 11.45% [20]. Hence, a clear understanding of all root canals is required to enhance the success rate of root canal treatment.

The success in this reported case can be attributed to the fact that we found that the lower third of the root canal of the tooth had mutated through detailed examination. When missing root canals were suspected during the treatment, we used CBCT to confirm the position of the root canals in a timely manner and identified five root canals to complete the treatment.

The bifurcation of the case was located on the lower third of the root canal. It was difficult to perform RCT because of poor vision and low brightness. The orifice of the root canal was then trimmed, and the root canals were examined by a dental operating microscope. A small quantity of calcium hydroxide paste (white) was placed on the dentin of the bifurcate position, where the reflection of light enhanced the vision in the root canal and helped locate the root canals.

The current literature usually reports the number of root canals of the first premolar on the mandible to be 1–4, and some canals have a type C roots. The differences in the shape and ratio of the root canal of the mandibular first premolar in different populations are small. According to the standard classification [8], statistical results show a similar overall trend in the root canal of the mandibular first premolar: most have one canal, a few have two root canals, and cases of three or more canals are rare and have only occasionally been reported. In addition to the abovementioned classification, there are cases (ca. 10–18%) of type C roots in the mandibular first premolar [21]. We have collated a number of case reports on the root canal morphology of the mandibular first premolar for nearly a decade, as detailed in Table 1 [19, 22–25]. According to the combination of many current research results, there are two or more root canals in the mandibular first premolar, both in the middle and the lower third of the root canal [26, 27]. Teeth with type C roots are generally accompanied by the presence of a radicular groove. There exist four types of subdivisions of type C roots, among which a semilunar buccal canal and a non-C-shaped lingual canal are most common [28].

**Table 1:** Summary of the published case reports on the root canal morphology of the mandibular first premolar

Ref no.	Age & Sex	No. of canals	Features of the root canals	type	Symmetry or not
[22]	24 years old, male	C-shape	a deep lingual groove C-shape (Category III)	TYPE 1	YES
[23]	19 years old, female	3	MB canal and DB canal and a-mid canal	TYPE 2	N/A
[24]	35 years old, female	3	MB canal and DB canal and lingual canal	TYPE 4	N/A
[25]	24 years old, male	4	MB canal, ML canal, DB canal, DL canal	TYPE 3	N/A
[19]	48 years old, female	4	MB canal, ML canal, DB canal, DL canal	TYPE 3	N/A

In the case of three mandibular premolar canals, the mesiobuccal canal from the middle of the canal is divided into two root canals at the apex of the canal, namely, the mesiobuccal canal and mesiobuccal-2 canal and an independent distobuccal canal. The mandibular premolars of the four canals often appear as a mesiobuccal root canal, mesiolingual root canal, distobuccal root canal, and distolingual root canal. To date, there is no report on the existence of five canals in the mandibular first premolar. To account for all root canal variations in the mandibular first premolar, we suggest that dental CBCT can be used for further diagnosis when X-ray fails to show a clear image of the lower 1/3 of the canal.

In conclusion, we report for the first time the clinical diagnosis and treatment of five root canals in the mandibular first premolar. This case advances the understanding of the variation in the anatomical structure of the mandibular first premolar and provides a reference for other similar cases.

## Data Availability

The datasets used and/or analyzed during the current study are available from the corresponding author upon reasonable request.

## Abbreviations

CBCT: Cone Beam Computed Tomography
RCT: Root Canal Therapy
EDTA: Ethylenediaminetetraacetic Acid
